# Association between veterinary technical practices, mastitis control, and milk quality in intensive dairy farms of Al-Kharj, Saudi Arabia

**DOI:** 10.3389/fvets.2026.1839108

**Published:** 2026-07-21

**Authors:** Darelglal A. G. Abdallah, Mohamed S. Abdellatif, Mona M. Sobhy, Harb A. E. H. El-Bardisy

**Affiliations:** 1 Department of Nursing, College of Applied medical sciences, Prince Sattam Bin Abdulaziz University, Al-Kharj, Saudi Arabia; 2 Department of Psychology, College of Education in Al-Kharj, Prince Sattam Bin Abdulaziz University, Al-Kharj, Saudi Arabia; 3 Department of Reproductive Diseases, Animal Reproduction Research Institute, Agriculture Research Center (ARC), Giza, Egypt; 4 Department of Agricultural Economics, Faculty of Agriculture, Al-Azhar University at Assiut, Assiut, Egypt

**Keywords:** disease surveillance, epidemiology, farm biosecurity, herd health management, livestock production systems, subclinical infection, udder health

## Abstract

**Introduction:**

Bovine mastitis is one of the most important diseases affecting dairy production worldwide, resulting in reduced milk yield, impaired milk quality, increased treatment costs, and substantial economic losses. This study evaluated the association between veterinary technical practices, mastitis occurrence, and milk quality in intensive dairy farms in Al-Kharj, Saudi Arabia.

**Methods:**

A cross-sectional survey was conducted between May and September 2024 involving 150 intensive dairy farms selected by stratified random sampling. Data were collected using structured questionnaires, farm health records, and on-farm observations. Variables included routine California Mastitis Test (CMT) implementation, hygiene and milk management scores, vaccination practices, technical staff qualifications, and mastitis occurrence. Associations were evaluated using Chi-square tests and multivariable logistic regression.

**Results:**

Mastitis was reported in 116 of 150 farms (77.3%), while the estimated animal-level occurrence of clinical mastitis was approximately 12%. Routine CMT implementation was associated with significantly lower odds of mastitis occurrence (OR = 0.46; 95% CI: 0.24–0.88; *p* < 0.05). Farms lacking qualified technical personnel had higher odds of mastitis occurrence (OR = 2.15; 95% CI: 1.12–4.13; *p* < 0.05). Only 28.0% of farms practiced mastitis vaccination, and low vaccination uptake was associated with increased mastitis occurrence (OR = 1.89; 95% CI: 1.01–3.52). Hygiene score was not significantly associated with mastitis occurrence or milk quality (*p* > 0.05).

**Discussion:**

The findings demonstrate that routine diagnostic screening and qualified veterinary technical support play key roles in mastitis control in intensive dairy systems. Strengthening preventive herd health programs, improving technical capacity, and promoting early detection strategies are essential to enhance udder health, improve milk quality, and support sustainable dairy production under arid environmental conditions.

## 1 Introduction

Bovine mastitis is still a big health and economic problem for dairy farmers all over the world. It has a big impact on milk production, composition, and the overall profitability of the farm. Mastitis is associated with increased somatic cell counts, reduced milk quality, higher treatment costs, increased culling rates, and greater labor requirements in dairy herds (1). Recent comprehensive reviews underscore mastitis as a persistent constraint on global dairy industry sustainability and advocate for integrated prevention and control strategies that span diagnostics, hygiene, and herd health management (2, 3).

The economic burden of mastitis is well documented, affecting both developed and emerging dairy sectors. Control strategies with proven impacts include routine screening, milking hygiene, vaccination, and improved farm-level management practices that collectively reduce pathogen pressure and infection incidence (4). Reviews emphasize the importance of on-farm diagnostics such as the California Mastitis Test (CMT) and on-farm culture systems in early detection and management of subclinical mastitis (5, 6).

Routine diagnostic screening plays a critical role in timely identification of udder infections before clinical signs emerge. The CMT remains widely used as a rapid cow-side tool, enabling early intervention that can mitigate disease progression and reduce reliance on therapeutic antibiotics, an approach increasingly emphasized in global mastitis control frameworks due to concerns over antimicrobial resistance (5).

Operational and management factors significantly influence mastitis dynamics. Studies in diverse dairy systems have shown that inadequate milking routines, inconsistent hygiene practices, and limited preventive measures contribute to higher mastitis prevalence and poorer milk quality outcomes. For instance, a recent field survey in Egyptian dairies highlighted that variable management practices, including inconsistent use of diagnostic approaches and hygiene standards, were associated with mastitis occurrence and milk quality issues (7).

Despite advances in mastitis diagnostics and prevention internationally, there is limited quantitative evidence assessing how routine veterinary technical practices such as frequency of screening, hygiene management, and preventive vaccination are implemented in intensive dairy clusters of the Middle East. Specifically, in Al-Kharj, Saudi Arabia, large commercial farms operate under arid climatic conditions that may exacerbate udder health challenges due to heat stress and environmental pathogens. Understanding the interplay between farm-level technical practices and udder health outcomes is essential to guide targeted control measures under local conditions.

Therefore, this study aimed to evaluate the implementation of routine veterinary technical practices and to investigate their association with mastitis occurrence and milk quality in intensive dairy farms of Al-Kharj, Saudi Arabia. By identifying key gaps in preventive technical management, the findings offer evidence to support improved mastitis control efforts in intensive production systems.

## 2 Materials and methods

### 2.1 Study design and study area

A cross-sectional study was conducted between May and September 2024 in Al-Kharj, Saudi Arabia, one of the largest and most intensive dairy production regions in the country. The area hosts a high concentration of commercial dairy farms, characterized by large herd sizes, advanced production systems, and year-round milk production under arid climatic conditions.

Al-Kharj is considered a major dairy hub, with more than 400 registered intensive dairy farms operating within a relatively lack of limitation geographic area, resulting in a high farm density. Farms are typically organized in clustered production zones, which may facilitate the transmission of infectious diseases such as mastitis due to increased animal movement, shared resources, and environmental contamination. Al-Kharj is one of the most important dairy production regions in Saudi Arabia and hosts a high concentration of intensive commercial dairy farms. Although ambient temperatures frequently exceed 45 °C during summer, dairy production is sustained through the widespread use of modern management practices and environmental control technologies, including climate-controlled housing, evaporative cooling systems, mechanical ventilation, shade structures, and optimized feeding programs. In arid environments, these measures enable high-yielding dairy animals stay productive while reducing heat stress. As a result, despite its difficult climate, the area has emerged as a significant dairy production hub (8). Al-Kharj is a suitable location for assessing the effects of veterinary technical methods on the incidence of mastitis and milk quality because of its high farm density, intensive production, and difficult climatic conditions.

### 2.2 Study population

The study population consisted of all intensive dairy farms registered with the Ministry of Environment, Water, and Agriculture (MEWA) in Al-Kharj, Saudi Arabia, during the study period. Eligible farms were those engaged in commercial milk production under intensive management systems and maintaining farm-level veterinary health records.

Farms were included if they met the following criteria: active dairy production during the study period, availability of farm health and production records for at least the previous 6 months, and willingness of farm owners or managers to participate in the study. Farms lacking reliable health records or operating under extensive or smallholder systems were excluded.

### 2.3 Sampling frame and sample size determination

The sampling frame was obtained from the official registry of (MEWA), which listed 428 eligible intensive dairy farms in Al-Kharj during the study period.

The initial minimum sample size was estimated using the standard formula for prevalence studies (Equation 1). Because the total number of registered dairy farms in the study area was finite (approximately 400 farms), the calculated sample size was subsequently adjusted using the finite population correction formula (Equation 2).

The required sample size was initially estimated using Cochran's formula (9) for proportions:


$$\begin{array}{l} n_{0}=\;Z^{2}P\;\left(1-P\right)/d^{2} \end{array}$$ (1)

where *Z* is the standard normal deviate corresponding to a 95% confidence level (1.96), *P* is the expected prevalence (0.50), and *d* is the desired precision (0.05). Based on these assumptions, the initial sample size was 384 farms.

Because the target population of registered intensive dairy farms in Al-Kharj was finite (*N* = 428), a finite population correction (FPC) was applied according to the following equation:


$$\begin{array}{l} n\;=\;n_{0}/\left[1\;+\;\left(n_{0}-1\right)/N\right] \end{array}$$ (2)

Application of the correction yielded a minimum required sample size of approximately 203 farms.

A total of 150 farms were ultimately included in the study. The reduction from the calculated sample size was attributable to practical constraints, including farm accessibility, incomplete farm records, and non-participation of some eligible farms. Nevertheless, the final sample represented approximately 35% of the total registered dairy farms in the region and provided sufficient statistical power for the planned analyses.

### 2.4 Sampling strategy

A stratified random sampling design was used based on herd size (small, medium, large) and production system. Farms were randomly selected within each stratum using computer-generated random numbers with proportional allocation.

### 2.5 Data collection and variable definition

Data were collected through structured questionnaires, direct observation, and review of farm veterinary records.

### 2.6 Hygiene and milk management scoring system

Farm hygiene and milk management practices were evaluated using a structured observational scoring system adapted from previously validated dairy hygiene assessment frameworks (2, 7). The assessment comprised five domains, including milking hygiene, udder preparation, equipment sanitation, housing cleanliness, and milk handling and storage practices, which are widely recognized as critical determinants of mastitis risk and milk quality. The hygiene and milk management scoring system used for farm assessment is presented in Supplementary Table S1.

Each domain consisted of specific observable indicators assessed during on-site visits. Individual items were scored using a three-point ordinal scale (0 = poor, 1 = moderate, and 2 = good), a method commonly applied in farm-level epidemiological studies to capture gradations in management quality while maintaining simplicity and reproducibility (7). The scores were summed to generate a composite hygiene and milk management score ranging from 0 to 20.

For analytical purposes, farms were categorized into four levels based on their total score: poor (0–5), average (6–10), good (11–15), and excellent (16–20). These thresholds were selected to reflect meaningful differences in management quality and to ensure balanced group distribution, consistent with approaches used in similar dairy management studies. This categorization facilitates comparison between farms and allows for interpretation of management practices in relation to mastitis occurrence and milk quality (2).

#### 2.6.1 Hygiene score categorization

The composite hygiene and milk management score was initially classified into four categories (poor, average, good, and excellent). For regression analysis, categories were combined into two groups because of the limited number of farms within some categories and to ensure sufficient observations per category for stable model estimation. This approach has been used in epidemiological studies when sparse cell counts may affect the validity of regression coefficients. Nevertheless, the original four-category classification was retained in the descriptive analyses (Table 1).

**Table 1:** Hygiene and milk management scoring checklist.

Domain	Indicator	Score 0 (poor)	Score 1 (moderate)	Score 2 (good)
Milking hygiene	Hand hygiene	No hand washing	Irregular washing	Consistent washing + gloves
	Milking procedure	Poor routine	Partially consistent	Standardized routine
Udder preparation	Pre-milking cleaning	Not performed	Inconsistent	Always performed
	Post-milking teat dip	Not used	Occasional use	Routine use
Equipment sanitation	Cleaning frequency	Rare cleaning	Daily	After each milking
	Disinfection	Not applied	Partial	Full sanitation
Housing	Bedding condition	Dirty/wet	Moderately clean	Clean/dry
	Manure removal	Irregular	Daily	Frequent/controlled
Milk handling	Storage hygiene	Poor	Acceptable	Hygienic
	Cooling system	Absent	Inconsistent	Proper cooling system

### 2.7 Vaccination assessment

Vaccination practices were evaluated through farm health records and interviews with farm managers. Information was collected regarding routine vaccination against major infectious diseases, including foot-and-mouth disease (FMD), brucellosis, and mastitis. Farms reporting mastitis vaccination primarily used commercially available polyvalent vaccines targeting major mastitis-associated pathogens, including *Staphylococcus aureus, Escherichia coli*, and other environmental coliform bacteria. Vaccination schedules were implemented according to manufacturer recommendations and veterinary guidance, typically involving primary vaccination followed by booster doses during the dry period or at designated intervals within herd health programs (10).

### 2.8 Diagnostic practices (CMT use)

Diagnostic practices were evaluated based on the implementation of (CMT), a widely accepted and cost-effective screening tool for detecting subclinical mastitis (5). Farms were classified as routine users if CMT was conducted regularly, defined as at least once per month or as part of routine herd health monitoring. Farms that performed CMT sporadically or did not use the test were categorized as non-routine users. This classification reflects differences in disease surveillance intensity and is consistent with previous studies examining the role of early detection in mastitis control (11).

### 2.9 Technical staff qualification

Technical capacity was assessed based on the presence of trained veterinary personnel or qualified herd health managers responsible for disease diagnosis, treatment, and preventive program implementation. Farms were categorized as having adequate technical expertise if such personnel were consistently involved in herd health management, and as lacking technical expertise if management decisions were made without professional veterinary support. This classification is supported by evidence indicating that veterinary involvement is a key determinant of disease control efficiency in dairy systems (6).

### 2.10 Mastitis case definition

Mastitis occurrence was defined at farm level as the presence of at least one clinically diagnosed case within the previous 6 months. Clinical mastitis was identified based on standardized veterinary field criteria, including abnormal milk (clots, discoloration), udder inflammation (swelling, heat, pain), and/or systemic signs as recorded by attending veterinarians.

A farm was classified as mastitis-positive when at least one clinically or subclinical diagnosed mastitis case had been recorded during the 6 months preceding the survey. Diagnosis was based on veterinary clinical examination, farm health records, milk abnormalities, udder inflammation, and/or positive CMT results. Cases associated with confirmed FMD outbreaks and characterized primarily by vesicular lesions of the udder or teats were excluded from the mastitis classification to minimize disease misclassification (12).

### 2.11 Statistical analysis

Data were analyzed using SPSS. Descriptive statistics summarized farm characteristics.

Univariable logistic regression was used to screen variables, and those with *p* < 0.20 were included in multivariable logistic regression. A backward stepwise approach was applied while retaining biologically relevant variables.

Confounding was assessed by ≥20% change in regression coefficients. Interaction terms (e.g., hygiene × CMT use) were tested. Missing data (< 5%) were handled using complete case analysis. Residual and influence diagnostics, significance was set at *p* < 0.05.

## 3 Results

### 3.1 Farm demographic and occupational characteristics

The survey covered 150 dairy farms in Al-Kharj. Of them, 20 (13.3%) and 14 (9.3%) were principally goat and camel farms, respectively, and 116 (77.3%) were specialized cattle operations. In terms of managerial education, 48 supervisors (32.0%) had completed secondary school, 59 supervisors (39.3%) had completed basic or non-formal education, and 43 supervisors (28.7%) had completed university-level education. In terms of professional experience, 85 managers (56.7%) had fewer than 10 years, while 65 managers (43.3%) had more than 10.

The distribution of farm characteristics, including livestock focus, managerial qualification, and farming experience, is presented in Table 2.

**Table 2:** Socio-technical profile of surveyed farms (*n* = 150).

Variable	Category	*N*	Percentage (%)
Livestock focus	Cattle	116	77.3
	Goats	20	13.3
	Camels	14	9.3
Managerial qualification	University	43	28.7
	Secondary	48	32.0
	Basic	59	39.3
Experience	>10 years	65	43.3
	≤ 10 years	85	56.7

### 3.2 Mastitis occurrence and diagnostic practices

A farm-level mastitis occurrence of 77.3% was observed, with 116 of the 150 surveyed farms reporting at least one mastitis case during the 6 months preceding data collection. Among affected farms, the mean number of mastitis cases was 16.5 ± 12.8 cases per farm. Routine implementation of CMT was reported by 68 farms (45.3%), whereas 82 farms (54.7%) relied primarily on clinical observation without systematic screening (Table 3).

**Table 3:** Veterinary technical practices and mastitis outcomes.

Indicator	Value	Standard deviation (±)	Frequency (*n*)
Mastitis occurrence	77.3%	–	116
Mean cases per affected farm	16.5%	12.8	–
Routine California mastitis test implementation	45.3%	–	68
Treatment success rate	75.5%	9.1	–

Based on farm health records, the mean treatment success rate among farms reporting mastitis cases was 75.5% ± 9.1%. These findings indicate substantial variation in mastitis monitoring and control practices among intensive dairy farms in the study area.

### 3.3 Animal-level occurrence of clinical mastitis

Based on farm health records, the overall animal-level occurrence of clinical mastitis was 12.0% during the study period. This finding indicates that approximately one in every eight dairy animals experienced at least one clinically diagnosed mastitis episode. Although the study primarily focused on farm-level management practices, the observed animal-level occurrence highlights the substantial burden of mastitis within intensive dairy production systems in the study area.

### 3.4 Hygiene rating and milk quality acceptance

Farms were categorized into four hygiene levels: excellent (18.7%), good (36.0%), average (31.3%), and poor (14.0%; Table 4).

**Table 4:** Association between hygiene rating and milk quality acceptance.

Hygiene	Total	Accepted	Percentage (%)
Excellent	28	17	60.7
Good	54	35	64.8
Average	47	33	70.2
Poor	21	13	61.9

Milk quality acceptance rates were:

Chi-square analysis showed no statistically significant association between hygiene rating and milk quality acceptance (*p* = 0.832).

### 3.5 Preventive vaccination adherence

Vaccination adherence varied according to vaccine type:

- Foot-and-mouth disease (FMD): 98 farms (65.3%).
- Brucellosis: 76 farms (50.7%).
- Mastitis-specific vaccination: 42 farms (28.0%).

The difference in adherence between FMD vaccination and mastitis-specific vaccination was statistically significant (*p* < 0.001; Table 5).

**Table 5:** Vaccination adherence rates (*n* = 150).

Vaccine	Adherence %	*N*
FMD	65.3	98
Brucellosis	50.7	76
Mastitis	28.0	42

### 3.6 Reported operational barriers

Farm managers identified several operational constraints affecting mastitis control and preventive practice implementation (Table 6).

**Table 6:** Reported barriers to effective mastitis control.

Rank	Barrier type	Frequency (*n*)	Percentage (%)
1	High medication costs	124	82.7
2	Technical expertise gap	98	65.3
3	Diagnostic delays (no CMT)	85	56.7
4	Economic fluctuations	76	50.7

### 3.7 Multivariable logistic regression analysis

Multivariable logistic regression analysis was performed to identify factors associated with bovine mastitis occurrence at the farm level.

Routine implementation of (CMT) was associated with reduced odds of mastitis (OR = 0.46; 95% CI: 0.24–0.88; and *p* = 0.019). Farms with limited technical expertise had higher odds of mastitis occurrence (OR = 2.15; 95% CI: 1.12–4.13; and *p* = 0.021). Lack of mastitis-specific vaccination was also associated with increased risk (OR = 1.89; 95% CI: 1.01–3.52; and *p* = 0.045).

Hygiene score was not significantly associated with mastitis occurrence (OR = 1.08; 95% CI: 0.67–1.74; and *p* = 0.832). Diagnostic delay showed a borderline association (OR = 1.76; 95% CI: 0.95–3.24; and *p* = 0.071). High medication cost was associated with increased mastitis risk (OR = 2.48; 95% CI: 1.29–4.76; *p* = 0.006).

The model showed acceptable fit (Hosmer–Lemeshow test, *p* > 0.05) and no multicollinearity (VIF < 5; Table 7).

**Table 7:** Multivariable logistic regression analysis of factors associated with bovine mastitis occurrence in dairy farms (*n* = 150).

Variable	Category	OR (adjusted)	95% CI	*p*-value
CMT implementation	Routine use	0.46	0.24–0.88	0.019^*^
	No routine use (reference)	1.00	–	–
Technical expertise	Adequate	1.00	–	–
	Limited expertise	2.15	1.12–4.13	0.021^*^
Mastitis vaccination	Implemented	1.00	–	–
	Not implemented	1.89	1.01–3.52	0.045^*^
Hygiene score	High (excellent/good)	1.00	–	–
	Low (average/poor)	1.08	0.67–1.74	0.832
Diagnostic delay (no CMT)	Present	1.76	0.95–3.24	0.071
	Absent	1.00	–	–
Medication cost constraint	High	2.48	1.29–4.76	0.006^*^

^*^Indicates a statistically significant association at *p* < 0.05.

## 4 Discussion

This study evaluated the association between veterinary technical practices and the occurrence of bovine mastitis, as well as their implications on milk quality in intensive dairy farms. The findings demonstrate that mastitis remains highly prevalent (77.3%) and is strongly influenced by gaps in preventive management, particularly diagnostic screening, technical expertise, and vaccination practices. These results highlight persistent challenges in implementing effective herd health strategies in intensive production systems, especially under arid environmental conditions.

### 4.1 Mastitis occurrence and diagnostic practices

The high prevalence of mastitis observed in this study confirms that udder health remains a critical constraint in intensive dairy systems. The mastitis occurrence observed in this study (77.3%) is higher than many reports from international dairy systems, where prevalence typically ranges between 30 and 60%, reflecting differences in management practices, environmental conditions, and diagnostic intensity (2, 3). Environmental stressors, including high ambient temperatures typical of arid regions, may exacerbate susceptibility to infection by impairing immune function and increasing pathogen exposure.

A key finding of this study is the significant protective effect of routine use of the California Mastitis Test, which was associated with reduced odds of mastitis occurrence. This supports recent evidence emphasizing that early detection of subclinical infections is fundamental to effective mastitis control. Contemporary studies have demonstrated that routine screening not only reduces disease progression but also minimizes antibiotic use and improves milk quality outcomes (5). Furthermore, emerging research in 2024–2025 highlights the growing role of precision livestock technologies and predictive analytics in enhancing early mastitis detection, reinforcing the importance of integrating routine diagnostic tools within modern herd management systems (6).

Although diagnostic delay was not significantly associated with mastitis occurrence (*p* = 0.071), the magnitude and direction of the observed effect suggest that delayed diagnosis may contribute to increased disease risk. Because the confidence interval included the null value and the association did not meet the predefined significance criterion, this finding should be interpreted cautiously (13). Further studies with larger sample sizes are needed to determine whether this observed trend represents a true association.

The observed animal-level occurrence of clinical mastitis (12.0%) represents a noteworthy finding with important implications for dairy production and animal health. Clinical mastitis is associated with reduced milk yield, impaired milk quality, increased treatment costs, elevated labor requirements, and premature culling, all of which contribute to substantial economic losses for dairy producers. In addition, mastitis negatively affects animal welfare through pain, inflammation, and reduced productive performance (14).

The occurrence observed in the present study is comparable to values reported in several intensive dairy production systems, although considerable variation exists among regions due to differences in management practices, herd size, environmental conditions, and diagnostic criteria (1). Previous studies have reported animal-level clinical mastitis occurrence ranging from approximately 5 to 20% in intensive dairy herds, placing the present findings within the range reported internationally. The relatively high farm-level occurrence (77.3%) combined with a 12.0% animal-level occurrence suggests that mastitis remains widely distributed across dairy farms in the study area, even when only a proportion of animals within each herd are affected.

These findings underscore the importance of strengthening mastitis prevention and control programs, particularly through routine diagnostic screening, improved milking management, staff training, and implementation of herd health monitoring programs. Given the economic and welfare consequences of mastitis, reducing animal-level occurrence should remain a priority for dairy producers and veterinary authorities in intensive production systems (15).

### 4.2 Milk hygiene score associated with mastitis

Contrary to expectations, hygiene score was not significantly associated with mastitis occurrence in the present study. This finding differs from numerous reports demonstrating that inadequate hygiene increases the risk of both contagious and environmental mastitis by increasing bacterial exposure during milking and within the housing environment (1). Several explanations may account for this discrepancy. First, most farms included in the study operated under intensive commercial management systems and generally maintained moderate to good hygiene standards, resulting in limited variability among farms. Such homogeneity may have reduced the ability of the analysis to detect statistically significant differences (5).

Second, the hygiene score was derived from a single observational assessment conducted during the study period. Although standardized criteria were used, the score may not have fully captured temporal variations in hygiene practices or specific management deficiencies occurring before the onset of mastitis cases (10). Consequently, some degree of exposure misclassification cannot be excluded. Third, mastitis is a multifactorial disease influenced by numerous interacting factors, including milking procedures, pathogen pressure, housing conditions, stocking density, nutrition, cow immunity, and the quality of veterinary supervision. The significant effects observed for routine diagnostic screening and technical expertise in the present study suggest that these factors may have exerted a stronger influence on mastitis occurrence than hygiene score alone (11).

Similar findings have been reported in some herd-level studies where hygiene indicators were not independently associated with mastitis after adjustment for other management variables, despite their established biological importance (4). In contrast, several studies conducted in Europe, North America, and developing dairy systems have demonstrated significant associations between poor hygiene and increased mastitis risk, particularly under conditions of inadequate milking hygiene, poor bedding management, or high environmental pathogen loads (6). Therefore, the absence of a statistically significant association in the present study should not be interpreted as evidence that hygiene is unimportant. Rather, it may reflect the relatively uniform management conditions of the surveyed farms, limitations of the scoring methodology, or the influence of other herd-level factors that were not fully captured in the analysis.

### 4.3 Technical expertise and herd health management

The present study identified limited technical expertise as a significant risk factor for mastitis occurrence, indicating that farms with inadequately trained personnel are more vulnerable to disease outbreaks. This finding is consistent with previous research demonstrating that the effectiveness of mastitis control programs is highly dependent on the knowledge and skills of farm workers (3, 16). Proper implementation of milking hygiene, early disease recognition, and adherence to treatment protocols require continuous training and supervision.

Recent literature further emphasizes that human factors are central to herd health outcomes, particularly in large-scale intensive systems where management errors can rapidly influence disease dynamics. In this context, strengthening technical capacity through targeted training programs and veterinary extension services is essential for improving mastitis control and overall farm productivity.

### 4.4 Vaccination and preventive strategies

The study revealed low adoption of mastitis-specific vaccination, which was associated with increased disease risk. While vaccination against mastitis causing pathogens remains variable in effectiveness due to pathogen diversity, recent reviews suggest that it can serve as a valuable complementary tool when integrated with other preventive measures (2, 3). The low uptake observed in this study may reflect economic constraints, limited awareness, and uncertainty regarding vaccine efficacy.

Recent advances highlight ongoing developments in mastitis vaccine research, including improved antigen targeting and immunomodulatory approaches aimed at enhancing protection against both contagious and environmental pathogens. However, adoption at the farm level remains inconsistent, indicating a need for clearer guidelines and cost-effective vaccination strategies.

### 4.5 Hygiene management and milk quality

Contrary to expectations, no significant association was observed between hygiene score and mastitis occurrence in the present study. This finding differs from numerous reports demonstrating that inadequate hygiene increases the risk of both contagious and environmental mastitis through greater exposure of the udder to pathogenic microorganisms (9).

A potential limitation of this study is the dichotomization of hygiene and milk management scores for multivariable analysis. Although this approach improved model stability and reduced sparse-data problems, it may have resulted in some loss of information and reduced sensitivity to detect graded associations between management quality and mastitis occurrence. Consequently, subtle differences among farms classified as poor, average, good, or excellent may not have been fully captured. Future studies involving larger populations should retain the original ordinal categories to evaluate potential dose-response relationships more comprehensively.

### 4.6 Several factors may explain this discrepancy

First, the study was conducted in intensive commercial dairy farms that generally maintained moderate to good hygiene standards, potentially limiting the variability required to detect statistically significant differences between farms (10).

Second, the hygiene assessment was based on a composite observational scoring system that may not have fully captured temporal variations in hygiene practices or specific risk factors directly related to mastitis transmission.

Third, mastitis occurrence is influenced by multiple interacting factors, including milking procedures, pathogen pressure, herd size, housing design, nutrition, immune status, and veterinary management practices, some of which may have confounded the relationship between hygiene and disease occurrence (1).

In addition, the cross-sectional study design limits the ability to establish temporal relationships between hygiene practices and mastitis development.

Similar findings have been reported in some studies where hygiene indicators were not independently associated with mastitis after adjustment for other management variables, suggesting that hygiene may act in combination with other herd-level factors rather than as an isolated determinant (11).

Nevertheless, the established importance of hygiene in mastitis prevention should not be disregarded, and continued emphasis on proper sanitation and milking hygiene remains essential for udder health management. Interestingly, no significant association was found between hygiene score and milk quality acceptance. This suggests that structural hygiene assessment alone may not adequately reflect effective mastitis control. Previous studies have shown that consistent milking routines, including proper udder preparation and post-milking teat disinfection, are more influential than infrastructure quality alone (17, 18).

Mastitis is widely recognized as a multifactorial disease influenced by complex interactions between host, pathogen, and environmental factors (19). Recent research underscores that behavioral compliance and routine consistency play a more decisive role than static hygiene conditions. The use of a standardized hygiene and milk management scoring system improves reproducibility; however, some degree of observer subjectivity cannot be entirely excluded.

### 4.7 Economic constraints and management decisions

Economic factors, particularly high medication costs, were identified as major barriers to effective mastitis control (20). Farms experiencing financial constraints were more likely to adopt reactive rather than preventive management strategies, increasing disease risk. This finding aligns with recent economic analyses demonstrating that mastitis imposes substantial financial burdens on dairy systems and that cost-related limitations hinder the adoption of preventive measures (21).

Additionally, growing concerns regarding antimicrobial resistance are driving a global shift towards preventive approaches rather than reliance on antibiotic treatments (20). Recent studies emphasize the importance of antimicrobial stewardship in dairy production, highlighting the need to reduce antibiotic usage through improved diagnostics, vaccination, and management practices (22).

### 4.8 Implications for mastitis control in intensive systems

The findings of this study underscore the importance of adopting an integrated approach to mastitis control in intensive dairy systems. Routine diagnostic screening, particularly through CMT, should be institutionalized as a standard practice. Equally important is the development of technical capacity among farm personnel to ensure proper implementation of preventive measures (23).

In arid environments, where climatic stress may exacerbate disease susceptibility, additional strategies such as environmental management, heat stress mitigation, and enhanced biosecurity should be incorporated into herd health programs (21). The integration of modern technologies, including sensor-based monitoring and data-driven decision-making tools, may further enhance mastitis control in large-scale operations (24).

## 5 Study limitations

This study has several limitations that should be considered when interpreting the findings. The cross-sectional design limits the ability to establish causal relationships between veterinary practices and mastitis occurrence. Additionally, reliance on farm-level clinical records may introduce reporting bias, and the absence of bacteriological confirmation limits pathogen-specific interpretation. Milk quality assessment based on processing unit feedback rather than standardized laboratory analysis may also affect consistency. Future longitudinal studies incorporating microbiological diagnostics and repeated measurements are recommended to validate these findings and provide deeper insights into mastitis epidemiology.

## 6 Future perspectives

Future research should focus on longitudinal monitoring of dairy herds to evaluate the long-term impact of preventive interventions, including routine diagnostics, vaccination, and training programs. Economic evaluations of mastitis control strategies are also needed to support cost-effective decision-making at the farm level. Moreover, integrating emerging technologies such as artificial intelligence and precision livestock farming tools may offer new opportunities for early disease detection and improved herd health management.

## 7 Conclusion

Mastitis remains a significant challenge in intensive dairy production systems in Al-Kharj, Saudi Arabia, with a high farm-level occurrence observed among the surveyed farms. The study demonstrated that routine implementation of CMT and the availability of qualified technical personnel were associated with reduced mastitis occurrence, highlighting the importance of early disease detection and effective herd health management. In contrast, inadequate adoption of preventive measures, including mastitis vaccination and systematic monitoring, may contribute to the persistence of the disease within dairy herds.

The observed animal-level occurrence of clinical mastitis underscores the substantial implications of mastitis for milk production, milk quality, farm profitability, and animal welfare. Although no significant association was detected between hygiene score and mastitis occurrence, this finding should be interpreted cautiously in light of the study design and measurement limitations.

Overall, the findings emphasize the need to strengthen preventive veterinary services, enhance technical training, expand routine diagnostic screening programs, and promote evidence-based mastitis control strategies in intensive dairy farms. Such interventions may contribute to improved udder health, enhanced milk quality, and greater sustainability of dairy production under arid environmental conditions.

## Data Availability

The raw data supporting the conclusions of this article will be made available by the authors, without undue reservation.
