# Supplementary material for: Association between veterinary technical practices, mastitis control, and milk quality in intensive dairy farms of Al-Kharj, Saudi Arabia
Source: Front Vet Sci. 2026 Jul 21;13:1839108. doi: 10.3389/fvets.2026.1839108 (PMC13433199; doi:10.3389/fvets.2026.1839108)
Supplement: Supplementary file 1 [file Table_1.DOCX]

**Table 1. Socio-technical profile of surveyed farms (n = 150)**

| Variable | Category | Frequency (n) | Percentage (%) | P-value |
| --- | --- | --- | --- | --- |
| Livestock focus | Cattle | 105 | 70.0 | – |
|  | Goats | 30 | 20.0 |  |
|  | Camels | 15 | 10.0 |  |
| Managerial qualification | University/Higher | 43 | 28.7 | 0.262 |
|  | Secondary | 48 | 32.0 |  |
|  | Basic/non-formal | 59 | 39.3 |  |
| Experience (years) | >10 years | 65 | 43.3 | 0.103 |
|  | ≤10 years | 85 | 56.7 |  |

**4.2 Mastitis occurrence and diagnostic practices**

**Table 2. Veterinary technical practices and mastitis outcomes**

| Indicator | Value | SD (±) | Frequency (n) |
| --- | --- | --- | --- |
| Mastitis occurrence | 77.3% | – | 116 |
| Mean cases per affected farm | 16.5 | 12.8 | – |
| Routine CMT implementation | 45.3% | – | 68 |
| Treatment success rate | 75.5% | 9.1 | – |

**4.3 Hygiene rating and milk quality acceptance**

**Table 3. Association between hygiene rating and milk quality acceptance**

| Hygiene rating | Total farms (n) | Accepted quality (n) | Acceptance rate (%) | P-value |
| --- | --- | --- | --- | --- |
| Excellent | 28 | 17 | 60.7 | 0.832 |
| Good | 54 | 35 | 64.8 |  |
| Average | 47 | 33 | 70.2 |  |
| Poor | 21 | 13 | 61.9 |  |

**4.4 Preventive vaccination adherence**

**Table 4. Vaccination adherence rates (n = 150)**

| Vaccine type | Adherence (%) | Frequency (n) | Non-adherence (%) | P-value |
| --- | --- | --- | --- | --- |
| FMD | 65.3 | 98 | 34.7 | <0.001 |
| Brucellosis | 50.7 | 76 | 49.3 |  |
| Mastitis-specific | 28.0 | 42 | 72.0 |  |

**4.5 Reported operational barriers**

**Table 5. Reported barriers to effective mastitis control**

| Rank | Barrier type | Frequency (n) | Percentage (%) |
| --- | --- | --- | --- |
| 1 | High medication costs | 124 | 82.7 |
| 2 | Technical expertise gap | 98 | 65.3 |
| 3 | Diagnostic delays (no CMT) | 85 | 56.7 |
| 4 | Economic fluctuations | 76 | 50.7 |

**4.6 Multivariable Logistic Regression Analysis**

**Table 5. Multivariable Logistic Regression Analysis of Factors Associated with Bovine mastitis Occurrence in Dairy Farms (n = 150)**

| Variable | Category | OR (Adjusted) | 95% CI | p-value | Interpretation |
| --- | --- | --- | --- | --- | --- |
| CMT implementation | Routine use | 0.46 | 0.24 – 0.88 | 0.019* | Protective factor |
|  | No routine use (reference) | 1.00 | – | – | – |
| Technical expertise | Adequate | 1.00 | – | – | Reference |
|  | Limited expertise | 2.15 | 1.12 – 4.13 | 0.021* | Increased risk |
| Mastitis vaccination | Implemented | 1.00 | – | – | Reference |
|  | Not implemented | 1.89 | 1.01 – 3.52 | 0.045* | Increased risk |
| Hygiene score | High (Excellent/Good) | 1.00 | – | – | Reference |
|  | Low (Average/Poor) | 1.08 | 0.67 – 1.74 | 0.832 | Not significant |
| Diagnostic delay (no CMT) | Present | 1.76 | 0.95 – 3.24 | 0.071 | Borderline risk |
|  | Absent | 1.00 | – | – | Reference |
| Medication cost constraint | High | 2.48 | 1.29 – 4.76 | 0.006* | Significant risk factor |
